# Serum protein layers on parylene-C and silicon oxide: Effect on cell adhesion

**DOI:** 10.1016/j.colsurfb.2014.12.020

**Published:** 2015-02-01

**Authors:** Evangelos Delivopoulos, Myriam M. Ouberai, Paul D. Coffey, Marcus J. Swann, Kevin M. Shakesheff, Mark E. Welland

**Affiliations:** a Nanoscience Centre, Department of Engineering, University of Cambridge, Cambridge CB3 0FF, UK; b School of Systems Engineering, University of Reading, Reading RG6 6AY, UK; c School of Physics and Astronomy, University of Manchester, Oxford Road, Manchester M13 9PL, UK; d Farfield, Biolin Scientific, 62 Wellington Road South, Stockport SK1 3SU, Cheshire, UK; e School of Pharmacy, University of Nottingham, University Park, Nottingham NG7 2RD, UK

**Keywords:** Parylene-C, Silicon oxide, Serum protein adsorption, Fibronectin, Cell adhesion, Biosensing technique

## Abstract

Among the range of materials used in bioengineering, parylene-C has been used in combination with silicon oxide and in presence of the serum proteins, in cell patterning. However, the structural properties of adsorbed serum proteins on these substrates still remain elusive. In this study, we use an optical biosensing technique to decipher the properties of fibronectin (Fn) and serum albumin adsorbed on parylene-C and silicon oxide substrates. Our results show the formation of layers with distinct structural and adhesive properties. Thin, dense layers are formed on parylene-C, whereas thicker, more diffuse layers are formed on silicon oxide. These results suggest that Fn acquires a compact structure on parylene-C and a more extended structure on silicon oxide. Nonetheless, parylene-C and silicon oxide substrates coated with Fn host cell populations that exhibit focal adhesion complexes and good cell attachment. Albumin adopts a deformed structure on parylene-C and a globular structure on silicon oxide, and does not support significant cell attachment on either surface. Interestingly, the co-incubation of Fn and albumin at the ratio found in serum, results in the preferential adsorption of albumin on parylene-C and Fn on silicon oxide. This finding is supported by the exclusive formation of focal adhesion complexes in differentiated mouse embryonic stem cells (CGR8), cultured on Fn/albumin coated silicon oxide, but not on parylene-C. The detailed information provided in this study on the distinct properties of layers of serum proteins on substrates such as parylene-C and silicon oxide is highly significant in developing methods for cell patterning.

## Highlights

• We studied how cell adhesion is affected by serum protein adsorbed on parylene-C.
• Serum proteins form distinct layers when adsorbed onto parylene-C or silicon oxide.
• Biosensing technique elucidates contrasting protein layer densities and thicknesses.
• Fibronectin supports cell adhesion on both surfaces.
• Albumin outcompetes fibronectin on parylene-C and vice versa on silicon oxide.

## Graphical abstract

**Figure:**
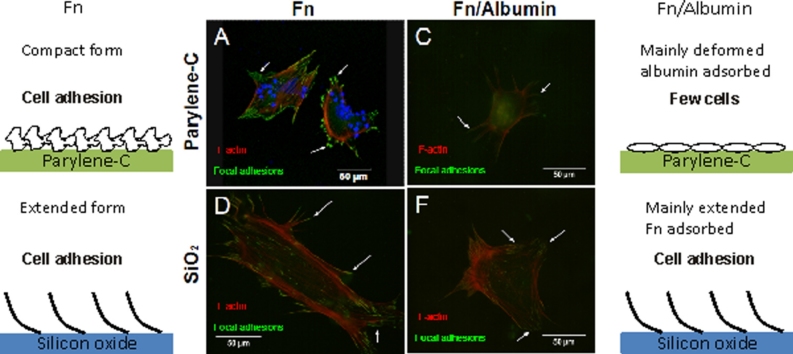

## 1 Introduction

Parylene is the trade name of a family of poly(*p*-xylylene) polymers, frequently used as insulators in microelectromechanical systems (MEMS) and electrode fabrication [1–3]. Parylene is applied on the desired surface via a chemical vapor deposition (CVD) process [4]. Recently, parylene has found numerous applications in bioengineering as an encapsulating material for medical implants [5], a stencil to pattern cells [6] and a physical trap for cells [7]. The most frequently used derivative of the parylene family is parylene-C (poly-monochloro-para-xylylene), in which an aromatic hydrogen atom is replaced by a chlorine atom. Parylene-C is known to be hydrophobic with a contact angle of approximately 100° [8]. Previous work has shown that parylene-C can facilitate protein adsorption and guide neuronal growth and neuronal network formation on parylene-C stripes deposited on a silicon oxide substrate [9]. Cell types that have been patterned with parylene-C include murine hippocampal neurons and glia [10,11], human teratocarcinoma (hNT) cell line derived neurons [12] and astrocytes [13], and HEK 293 cells [14].

Cell patterning techniques have considerable impact in tissue engineering, as they enable detailed studies of the extracellular microenvironment and may lead to directed stem cell differentiation [15]. However, in many cases the causal mechanisms of cellular guidance are unknown. For instance, the underlying material–protein and protein–cell interactions that promote cell adhesion on parylene-C still remain elusive. It is essential to identify and explain these interactions for two reasons. First, isolation of the necessary proteins involved in parylene-C cell patterning will allow one to avoid the use of serum with its inherent variability. Furthermore, such studies may enable the optimization of parylene–protein interactions toward the neuralization of progenitor cells, which is an area receiving strong interest.

Earlier experiments implicated the adsorption and conformation of serum glycoproteins, such as fibronectin (Fn) and vitronectin (Vn), in cell adhesion to a variety of substrates [16]. It is established that these serum proteins mediate cell adhesion by binding to transmembrane integrin receptors on the cells. The protein segment often involved in this interaction is the arginine–glycine–aspartic acid (RGD) peptide sequence and it is present in a number of extracellular matrix (ECM) proteins, including Fn and Vn [17]. Therefore, the orientation and folding of the adsorbed protein become critical factors in determining whether cultured cells will be able to interact with the RGD peptide and thus adhere to the surface. For example, even though adsorbed Fn has a higher surface density on poly(dimethylsiloxane) (PDMS) than on plasma treated PDMS, Fn promotes cell adhesion and growth only on the plasma treated PDMS [18]. The same effect was observed by Grinnell et al. where Fn was able to promote cell spreading only on hydrophilic and not hydrophobic substrates [19].

In this study, we describe the use of dual polarization interferometry (DPI) to quantify the adsorption process of serum albumin, fibronectin and a mixture of these two proteins on silicon oxide and parylene-C substrates. DPI is an optical biosensing technique that measures in real time the change in refractive index (RI) and thickness of thin layers formed on the top of a sensing waveguide [20]. This technique has been used to characterize in detail protein adsorption and changes in protein conformational state [21–24]. The aim of the present study is to show that these proteins (Fn and albumin) have different binding affinities and layer properties on the two substrates, due to the contrasting contact angles of silicon oxide and parylene-C, [8,25] and associate this to cell proliferation and growth. Indeed, our results demonstrate that after adsorption of the individual proteins, the layers formed have different structural properties depending on the substrate. Cultures of differentiated mouse embryonic stem cells (CGR8) on parylene-C and silicon oxide coated with protein highlight the effect of surface properties on cell adhesion and reinforce our model of the Fn and albumin structural state on these two substrates.

## 2 Materials and methods

### 2.1 Materials

Bovine serum albumin, HPLC grade water (resistivity > 18 MΩ cm), phosphate buffered saline (PBS) and sodium deodecyl sulphate (SDS) were purchased from Sigma (Sigma & Aldrich) and used as received. Bovine plasma Fibronectin was purchased from Life Technologies™. Parylene-C was purchased as a dimer from SCS™. Hellmanex® III (Hellma Analytics) (2% in deionized water) was used to clean the DPI injection loops. Untreated silicon oxynitride (SiON) multiple path length dual polarization interferometer (MPL-DPI) chips (Farfield-Biolin Scientific AB) are composed of approximately 11% of silicon nitride and 89% of silicon dioxide (SiO_2_). Comparison of surface coverage values of bovine serum albumin adsorbed onto SiON and SiO_2_ show no significant difference (Table S1) [26]. Silicon oxynitride DPI sensor surface and silicon dioxide surfaces are therefore referred as silicon oxide surfaces in the manuscript. Details of cleaning procedures can be found in our previous work [22]. As previously reported, contact angle measurements confirmed that DPI SiON and SiO_2_ surfaces have similar contact angles showing a good similarity of polarity [22].

### 2.2 Parylene-C coating

Prior to parylene-C coating, functionalization of the freshly UV/ozone cleaned SiON MPL-DPI chip and SiO_2_ substrate was performed to increase the hydrophobicity of the substrate in order to promote the formation of a stable parylene-C layer. The substrates were soaked for 1 h in a solution made of 4% (v/v) of trimethoxyphenylsilane in analar grade isopropanol, IPA. After rinsing with IPA the substrates were blown dry with a stream of nitrogen and cured in an oven at 110 ± 5 °C for 30 min. Following this surface modification, a 20–30 nm thick layer of parylene-C was deposited at room temperature on the treated substrates. A Labcoter 2 Parylene deposition Unit (Model PDS2010) was used to deposit the polymer at a rate of 1.298 nm per mg of dimer. The thickness of the deposited parylene-C layer was confirmed via ellipsometry, on an ellipsometer Nanofilm EP3, within 1 h of the deposition to minimize contamination. Different spots on three different samples were measured and the experimental data was fitted using a Cauchy model. Averages were drawn across the different samples. The deposition of the parylene-C layer was further confirmed by analyzing the surfaces using Atomic Force Microscopy (PicoPlus AFM with a PicoSPM II controller from Molecular Imaging, Agilent). Images were acquired at room temperature in air using the AC Mode with NSC36/no Al cantilevers (Mikromasch, force constant varying from 0.6 to 1.75 N/m).

### 2.3 DPI

A dual polarization interferometer (Farfield Analight® 4D, Biolin Scientific AB) was used to optically characterize the protein adsorption process on both unmodified silicon oxynitride and parylene-C coated sensor chips. The DPI sensor consists of two slab waveguides, one (buried) waveguide acts as a reference, while the second (exposed) is used to detect changes of the absorbed layer at the surface. When the light that propagates through the two waveguides exits the DPI sensor, the light from each waveguide interferes to create an interference pattern. The instrument alternately generates two orthogonal polarizations of light that excite the waveguide modes supported by the DPI sensor chip. These two orthogonally polarized waveguide modes are known as the transverse electric (TE) and transverse magnetic (TM) modes. Each mode has an evanescent field that exists within the sample and any changes occurring at the surface cause the velocity of the propagating mode to change. These changes are detected and measured experimentally as a change in the vertical position of the interference pattern, and is recorded in real time as TE and TM phase changes. The phase change of TE and TM signals are further analyzed by solving Maxwell's equations for a stack dielectric waveguide into two independent measurements which are the refractive index (RI) and thickness of the adsorbed layer [27]. The multiple path length dual polarization interferometer (MPL-DPI) is a modification of the DPI technology, with the same underlying principles, but with a new waveguide design which allows films coated outside of the instrument to be quantified. The experimental procedure and the method to calculate the refractive index and thickness of ex situ coated films using MPL-DPI was carried out according to a published protocol [28]. The instrument was set to operate at 20 °C. Details of the instrumentations can be found in our previous work [22].

### 2.4 Cell culture and immunohistochemistry

The mouse embryonic stem (mES) cell line CGR8 was obtained from Sigma (Sigma & Aldrich, UK). Pre-characterization of the CGR8 cell line was performed in our laboratory, which included the verification of stem cell marker expression and neuronal differentiation. The cells were kept in an undifferentiated state in LIF (Leukemia Inhibitory Factor) supplemented DMEM (Dulbecco's modified Eagle medium) media (10% fetal calf serum, 1% penicillin/streptomycin, 1% l-glutamine, 100 μM 2-mercaptoethanol). The mES cells were passaged and split (ratio 1:8) every 2 days. On day 0 mES cells were seeded on non-tissue culture treated petri-dishes at a density of 20,000 cells/mL and allowed to aggregate into embryoid bodies (EB) in advanced DMEM media (ADMEM/F12:neurobasal medium (1:1), 10% knockout serum replacement, 1% penicillin/streptomycin, 1% l-glutamine, 100 μM 2-mercaptoethanol) without LIF. Media was exchanged with fresh solution at day 2 and day 5 of differentiation. On day 6, EBs were collected, washed with PBS and resuspended in Trypsin/EDTA for 10 min at 37 °C. This process breaks down EBs and releases individual cells into the suspension. Trypsin was inactivated with aggregation medium and cells centrifuged at 180 × *g* for 5 min. The supernatant was then aspirated. Cells were resuspended in aggregation medium and then filtered through a 70 μm cell strainer on top of 50 mL centrifuge tube, in order to remove any large aggregates and matrix. The density of single cells in suspension was counted in a haemocytometer. Individual cells were seeded at 200 cells/mm^2^ on parylene-C and silicon oxide substrates that had been incubated for 2 h in Fn (50 μg/mL), albumin (5000 μg/mL) or Fn/albumin (50 and 5000 μg/mL) in PBS, at room temperature (20 °C). Thereafter the substrates were rinsed with PBS and incubated with cells at 37 °C. The effect of temperature on the amount of adsorbed proteins on silicon wafers was studied for albumin and fibronectin. The adsorbed amount of these proteins remains constant in the range of 20 °C to 40 °C showing no significant desorption [29].

The cultures were incubated for 3 days at 37 °C, 5% CO_2_, in advanced DMEM media that did not contain serum (ADMEM/F12:neurobasal medium (1:1), B27 supplement, 1% penicillin/streptomycin, 1% l-glutamine, 100 μM 2-mercaptoethanol). After 3 days in vitro (DIV) cell cultures were fixed in 4% paraformaldehyde (PFA) for 20 min and stained for *b*-tubulin III, *F*-actin and vinculin. Blocking against non-specific binding was performed for 1 h in 5% normal donkey serum for *b*-tubulin III or bovine serum albumin (BSA) for *F*-actin and vinculin staining. The primary antibody used was goat anti-Beta III tubulin at 1:100 dilution and was applied in donkey serum for 1 h at room temperature. The secondary antibody donkey anti-goat 594 Alexa Fluor™ was applied at 1:200 dilution in donkey serum for 1 h at room temperature. Phalloidin 594 Alexa Fluor™ and anti-vinculin 488 were applied in BSA for 1 h and 2 h at room temperature, at 1:100 and 1:250 dilutions, respectively. Substrates with stained cultures were mounted in Vectashield^®^ mounting medium with DAPI for nuclear labeling.

### 2.5 Image acquisition and analysis

Images were taken on a Leica confocal microscope with ×20 and ×40 lenses. Areas of dense cell populations were chosen. Images of *F*-actin and phalloidin stained cultures were subjected to a thresholding process, with the cut-off value carefully set for each image in order to include the maximum number of focal adhesion complexes, with as much detail possible, without including noise. From the histograms of pixel intensity, the total number of pixels corresponding to focal adhesions in a cell was calculated and compared to the total number of pixels in the specified cell, expressed as a percentage. For each protein coating on parylene-C and SiO_2_, percentages of focal adhesion complex areas were calculated from nine cells in four different substrates, across two independent experiments. Averages were compared across treatments and surfaces and were statistically analyzed with a one way ANOVA.

## 3 Results

### 3.1 Characterization of parylene-C layer

Parylene-C was coated using a chemical vapor deposition method on a silicon oxide chip surface pre-functionalized with phenylsilane in order to increase the adhesion stability of the film. This process results in the formation of a thin film of parylene-C that was characterized using the waveguide configuration of the MPL-DPI [28]. The MPL-DPI resolves the thickness and refractive index of ex situ coated ultrathin films to a high precision. MPL-DPI measured a thickness of 29.2 ± 0.3 nm and a RI of 1.653 ± 0.001 in good agreement with values expected according to the ex situ parylene-C coating procedure. The parylene-C layer was of sufficient thickness, so that these values could be further confirmed by a measurement with an ellipsometer for which a layer of 29.4 ± 0.4 nm in thickness and of 1.639 ± 0.001 in RI was fitted. Further analysis of the surfaces using atomic force microscopy before and after parylene-C coating confirms the deposition of a layer of parylene-C with a step height of approximately 30 nm (Fig. S1).

### 3.2 Protein adsorption analysis

**Fig. 1:**
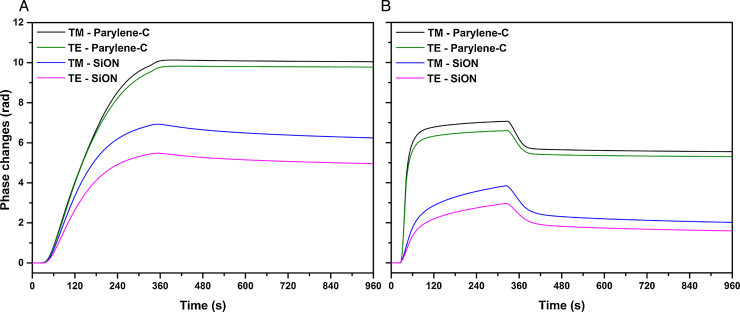
Real time TM and TE phase changes for adsorption of Fn at 50 μg/mL (A) and albumin at 5000 μg/mL (B) on parylene-C and SiON in PBS at 20 °C.

**Fig. 2:**
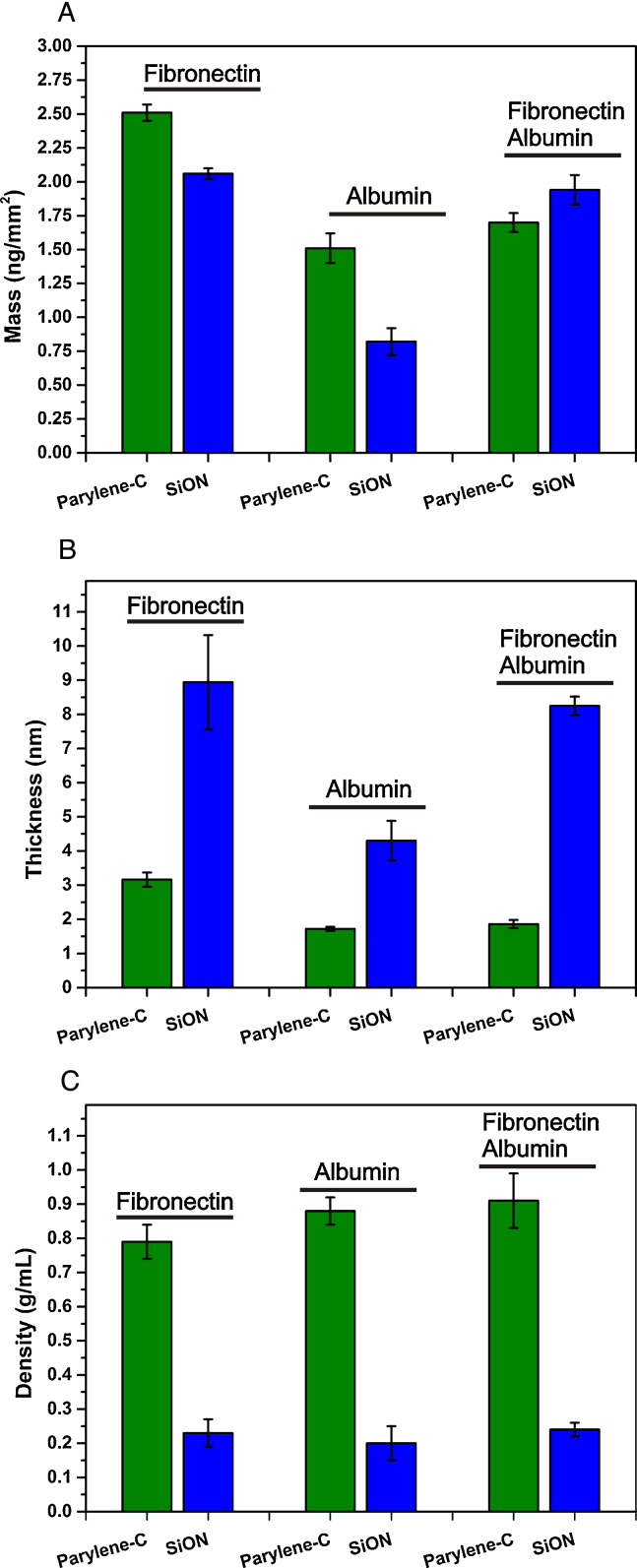
Surface coverage (ng/mm^2^) (A), thickness (nm) (B) and density (g/mL) (C) of adsorbed Fn (50 μg/mL), albumin (5000 μg/mL) and mixture of Fn/albumin (50 μg/mL/5000 μg/L) on parylene-C and SiON in PBS at 20 °C. Data is expressed as mean ± SD (*n* = 3).

The adsorption process of the serum proteins was examined at concentrations approximately 10 times lower than those found in serum, as most tissue cultures are carried out with media containing 10% serum [19,30,31]. The concentrations of serum proteins used were 50 μg/mL for Fn and 5000 μg/mL for albumin. The binary mixture of Fn/albumin was studied at the same concentrations resulting in a ratio of 1:100 (w/w) similar to the ratio found in serum. The adsorption process on the hydrophilic surface silicon oxide and on the hydrophobic surface parylene-C was studied using DPI by measuring the real time phase changes in the two polarizations, TM and TE as displayed in Fig. 1. The phase changes in TM and TE respond differently in accordance with Maxwell's equations and can be used to determine the surface coverage and structural properties of the protein layers, such as changes in density or conformation. At a qualitative level for isotropic layers, this can be observed in the raw data as a phase change of the TM and TE polarizations of light with a TM/TE ratio of approximately 1.3, signifying that the layer formed is very diffuse. On the other hand, a similar TM and TE phase change reflects the formation of a very dense layer (e.g. with a TM/TE ratio moving closer to 1.0). The adsorption process was monitored during a 5 min injection and during the rinsing phase. For albumin and Fn adsorption, a successive second injection was performed to reach saturation. Surface coverage, thickness, RI and density values were extracted at the maximum coverage reached during the injection (Fig. 2, Table S2) and after 5 min rinsing (Table S2).

### 3.3 Adsorption of Fn

Fn at 50 μg/mL in PBS showed more adsorption on parylene-C, compared to SiON, as reflected by the higher phase change response observed during the injection (Fig. 1A). The adsorbed mass values reached 2.51 ± 0.06 ng/mm^2^ on parylene-C and 2.06 ± 0.04 ng/mm^2^ on SiON (Fig. 2A, Table S2). On both substrates the surface was saturated during the first injection, as only a slight increase (of less than 5%) in surface coverage was monitored during the second injection (data not shown). The adsorption on parylene-C showed almost no reversibility (1% mass difference after 5 min rinsing) whereas more reversible binding was observed on SiON (10% mass difference after 5 min rinsing) (Table S2). From the TM/TE phase change ratios, we notice that the values are different on the two substrates, with TM/TE for parylene-C close to 1 and equal to 1.25 for SiON (Fig. 1A). These values suggest a significant difference in the structure of the layers formed, with a denser layer being formed on parylene-C and a more diffuse layer being formed on SiON. This is borne out, on analysis, as the resolved values obtained on parylene-C describe the formation of a thin and dense layer with a thickness of 3.2 ± 0.2 nm and a protein density of 0.79 ± 0.05 g/mL (Fig. 2B and C, Table S2). In contrast, the values obtained on SiON indicate the formation of a thick and diffuse layer with a thickness of 8.9 ± 1.4 nm and a density of 0.23 ± 0.04 g/mL (Fig. 2B and C, Table S2).

### 3.4 Adsorption of albumin

Similar to Fn, albumin showed significantly more binding on parylene-C compared to SiON with surface coverage values of 1.5 ± 0.1 ng/mm^2^ and 0.8 ± 0.1 ng/mm^2^, respectively (Fig. 2A, Table S2). On parylene-C the surface was rapidly saturated during the first injection as no increase of the surface coverage was observed during the successive second injection. However, on SiON, albumin adsorbs significantly slower and an increase in the surface coverage was observed after a second injection (15% mass increase, data not shown). The adsorption process is not reversible on parylene-C, while 20% of the mass absorbed is removed on SiON after 5 min rinsing (Table S2). The TM and TE phase changes observed during the injection is due to the adsorption of albumin and from the bulk change due to the high concentration of albumin used (Fig. 1B). However, a difference in TM/TE ratios between the two surfaces can be noticed with values of 1 for parylene-C and 1.3 for SiON. Similar conclusions to Fn can be drawn for the albumin layer structure on both substrates. A dense and thin layer is formed on parylene-C with a thickness of 1.72 ± 0.06 nm and a protein density of 0.88 ± 0.04 g/mL (Fig. 2B and C, Table S2). On SiON a diffuse and thick layer is formed with a thickness of 4.3 ± 0.6 nm and a protein density of 0.20 ± 0.05 g/mL (Fig. 2B and C, Table S2).

### 3.5 Adsorption of a Fn/albumin mixture at a 1:100 mass ratio

**Fig. 3:**
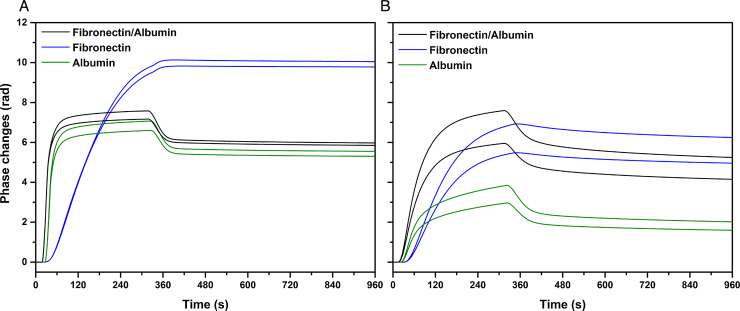
Real time TM and TE phase changes for adsorption on parylene-C (A) and SiON (B) of a mixture of Fn at 50 μg/mL and albumin at 5000 μg/mL compared with Fn at 50 μg/mL and albumin at 5000 μg/mL in PBS at 20 °C (injection start times are offset for clarity).

To mimic the conditions found in serum, the adsorption process of a mixture of Fn and albumin at a 1:100 mass ratio was studied. Strikingly, in this case, similar surface coverage is obtained on both substrates with mass values of 1.70 ± 0.07 ng/mm^2^ on parylene-C and slightly more, 1.9 ± 0.1 ng/mm^2^, on SiON (Fig. 2A, Table S2). As observed for individual protein solutions, two distinct layer structures are formed on the substrates. The real time phase change data, displayed in Fig. 3, reflect the difference of the formed layer structure with a TM/TE ratio of 1 on parylene-C and of 1.3 on SiON. The resolved thickness and density values show the formation of a thin and very dense layer on parylene-C (Th = 1.9 ± 0.1 nm, *d* = 0.91 ± 0.08 g/mL) and a thick and diffuse layer on SiON (Th = 8.2 ± 0.3 nm, *d* = 0.24 ± 0.02 g/mL) (Fig. 2B and C, Table S2). Both the phase changes and the values of the layer structure indicate that in both cases one protein is mainly adsorbed on each substrate. Indeed, we observe that on parylene-C the TM and TE changes in terms of kinetic and saturation values are almost identical to the albumin adsorption process (Fig. 3A). This is further highlighted by the structural analysis with mass, thickness and density values that are similar to the albumin layer, rather than the Fn layer (Fig. 2, Table S2). This result is consistent with a layer mainly composed of albumin. On SiON, the opposite observation is made. The TM and TE phase change kinetics and saturation values show high similarity to the Fn injection (Fig. 3B). A bulk contribution due to the high albumin concentration can be observed, however the surface coverage reached is close to the value for Fn adsorption and twice as much as albumin. The thickness and density values also indicate a layer structure similar to that obtained for just Fn. From these results we can conclude that on SiON, in contrast to parylene-C, Fn is mainly adsorbed. These results show that the co-incubation of albumin with Fn prevents the formation of a Fn layer on parylene-C, but not on SiON. In addition, no significant difference in the Fn layer properties can be noticed between the sole injection of Fn and the binary mixture Fn/albumin that would suggest a distinct conformational state.

### 3.6 Cell adhesion on Fn and albumin coated parylene-C and silicon oxide

In order to understand the effect of Fn and albumin layer properties on cell adhesion, we cultured differentiated CGR8 cells on parylene-C and SiO_2_ substrates that had been coated with Fn (50 μg/mL), albumin (5000 μg/mL) and a Fn/albumin mixture (50/5000 μg/mL). The CGR8 mouse embryonic stem cell line has been extensively used to generate a variety of cell types, such as cardiomyocytes [32] and dopaminergic neurons [33]. In this paper, we adapted a previously published protocol [34] to generate a mixed population of neurons and cells expressing fibroblast morphology. The presence of neurons is verified by immunofluorescent labeling of the neuronal marker *b*-tubulin III and allows us to draw comparisons to our previous study on patterning neurons on parylene-C [9]. The use of a cell line is also preferable to a primary cell culture, as the latter requires the use of animals and an ethical approval.

**Fig. 4:**
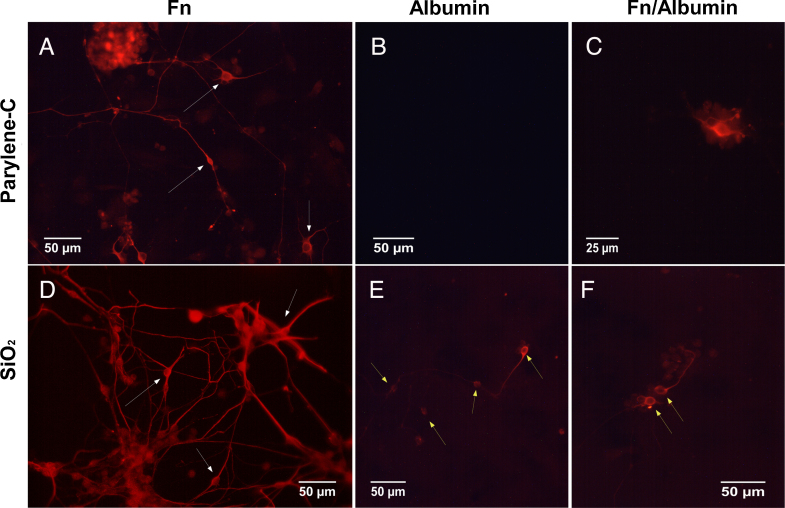
Representative field of views of neuronal populations from differentiated CGR8 cultures on protein treated parylene-C and SiO_2_ substrates. The red fluorescence denotes *b*-tubulin III on Fn (A), albumin (B) and Fn/albumin (C) coated parylene-C; Fn (D), albumin (E) and Fn/albumin (F) coated SiO_2_. Fn coated parylene-C and SiO_2_ hosted many neurons extending long processes (white arrows in (A) and (D)), whereas no cells were present on the albumin coated parylene-C (B). Neurons on Fn/albumin coated parylene-C were sparse and did not extend processes (C). Sparse populations of neurons were also found across albumin (E) and Fn/albumin (F) coated SiO_2_ but they have short neuronal processes (yellow arrows). (For interpretation of the references to color in this figure legend, the reader is referred to the web version of this article.)

SiO_2_ was used for the cell adhesion assay as protein adsorption on silicon oxynitride and silicon dioxide surfaces is very similar (Table S1) [21,26,35]. The cells were fixed at 3 DIV and stained either for *b*-tubulin III, for detection of neuronal subpopulations, or for *F*-actin/vinculin, for detection of actin filaments and vinculin (focal adhesions). Fig. 4 illustrates six representative fields of view from cultures on differently coated parylene-C and SiO_2_ substrates.

Overall, parylene-C and SiO_2_ coated with Fn hosted the richest neuronal populations, with neurons that extended multiple long processes (white arrows in Fig. 4A and D). In contrast, virtually no cells and very few neurons were detected on the albumin and Fn/albumin coated parylene-C, respectively (Fig. 4B and **C**). In addition, neurons on Fn/albumin coated parylene-C had few short processes, unlike the ones found on the Fn coated SiO_2_ and parylene-C substrates. On SiO_2_, neurons were identified across all the different treatments, Fn, albumin, Fn/albumin but neuronal populations were denser on Fn coated SiO_2_ (Fig. 4D). Furthermore, short neuronal processes were observed on albumin and Fn/albumin SiO_2_ substrates (yellow arrows in Fig. 4E and F).

**Fig. 5:**
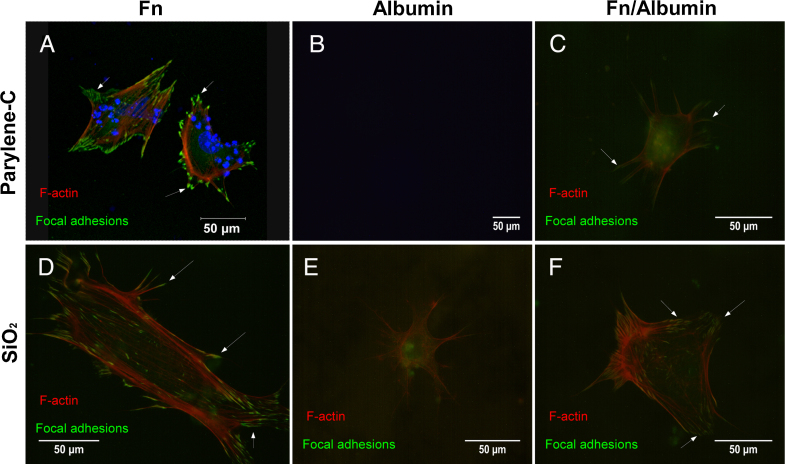
Fibroblast-like cells differentiated from mES cells (CGR8) on protein treated parylene-C and SiO_2_ substrates. Red, green and blue fluorescence denote *F*-actin, vinculin and nuclear (DAPI) staining, respectively on Fn (A), albumin (B) and Fn/albumin (C) coated parylene-C; Fn (D), albumin (E) and Fn/albumin (F) coated SiO_2_. Focal adhesions (white arrows) are more pronounced in Fn coated parylene-C and SiO_2_ (A and D) and appear as elongated areas of green fluorescence. No cells were detected on albumin coated parylene-C (B). On albumin coated SiO_2_ (E) vinculin is diffused throughout the cytoplasm, which denotes the absence of focal adhesions and lack of cell attachment to the ECM. The co-adsorption of Fn and albumin on parylene-C (C) and SiO_2_ (F) results in focal adhesion formation only on the SiO_2_, due to the absence of Fn from parylene-C.

**Fig. 6:**
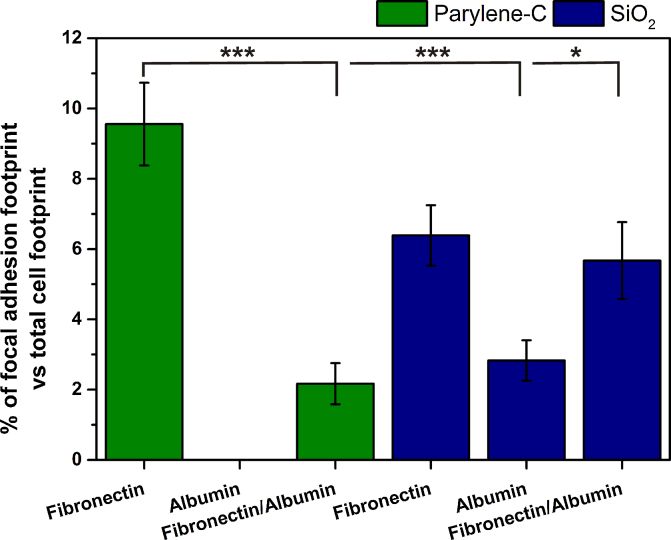
Percentage of total focal adhesion complex area out of total cell area, for each protein coated surface. On albumin coated parylene-C no cells were detected. Two independent experiments were conducted and averages were calculated from nine cells on four different substrates, for each condition and material. Error bars denote the standard error of means (SEM). One and three dots represent statistical significance of *P* ≤ 0.05 and *P* ≤ 0.001, respectively.

Integrin mediated attachment of cells to proteins adsorbed onto parylene-C and SiO_2_ treated substrates was assessed via vinculin and *F*-actin immunolabeling. When integrin receptors on the cell membrane recognize attachment sites on proteins in the ECM, they cluster into groups called focal adhesion complexes. Vinculin is then co-localized in these regions and initiates the polymerization of actin into *F*-actin microfilaments [36]. In Fig. 5A, D and F, focal adhesions can be distinguished as areas of intense green fluorescence (white arrows), located at the end of actin filaments, which appear as red striations. Image and statistical analysis reveal that on Fn coated parylene-C and SiO_2_, focal adhesion complexes occupy 9.6% ± 1.2% and 6.4% ± 0.9% of the total cell area, respectively (Fig. 6). Likewise, on Fn/albumin treated SiO_2_ focal adhesion coverage is 5.7% ± 1.1%. In contrast, when only albumin is adsorbed onto SiO_2_ or Fn competes with albumin on parylene-C, vinculin is diffused throughout the cellular soma and processes (diffuse green fluorescence in Fig. 5C and E). The percentage of focal adhesion areas drops to 2.2% ± 0.6% and 2.8 ± 0.6% for Fn/albumin coated parylene-C and albumin coated SiO_2_, respectively (Fig. 6). No cells were detected on albumin coated parylene-C (Fig. 5B).

The difference in percent focal adhesion area between Fn and Fn/albumin coated parylene-C is statistically significant (*P* ≤ 0.001). The same is true for Fn treated parylene-C and albumin (*P* ≤ 0.001) or Fn/albumin (*P* ≤ 0.05) treated SiO_2_. We hypothesize that on Fn treated parylene-C and on Fn, Fn/albumin treated SiO_2_, integrin receptors on the cell membrane can bind to the Fn adsorbed onto the surface and cluster into complexes. Vinculin is then localized and activated in the same regions. The opposite is true on Fn/albumin treated parylene-C and albumin treated SiO_2_, as integrin receptors on the cell membrane cannot locate ligands to bind to, in the ECM. The presence and absence of ligands on Fn and Fn/albumin treated parylene-C, respectively, highlights that Fn is outcompeted when it is co-adsorbed with albumin on a parylene-C. This effect however does not occur on Fn and Fn/albumin treated SiO_2_, as distinct focal adhesions form (Fig. 5D and F) and have equal footprints (Fig. 6) on both surfaces.

## 4 Discussion

### 4.1 Fibronectin layers

**Fig. 7:**
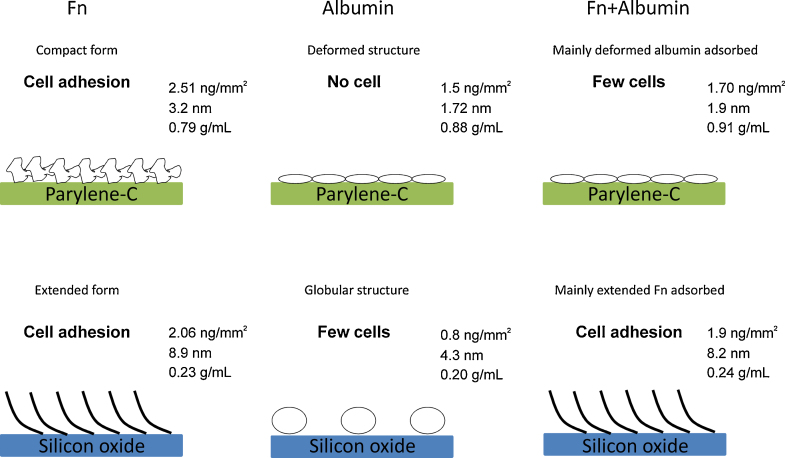
Proposed model of the layers formed by serum proteins adsorbed onto silicon oxide and parylene-C. Fn forms a dense and thin layer on parylene-C with proteins in a compact structure that promotes cell adhesion. On the opposite, on silicon oxide, a more diffuse and thicker layer is formed suggesting that Fn adopts an extended structure that also mediates cell adhesion. Albumin forms a dense and thin layer on parylene-C with protein molecules probably deformed, whereas a globular structure is observed on silicon oxide leading to the formation of a diffuse and thicker layer. The albumin coated parylene-C prevents cell adhesion, whereas few cells can grow on the albumin coated silicon oxide. Finally, the binary mixture of Fn and albumin results in the preferential adsorption of one of the protein, deformed albumin on parylene-C and extended Fn on silicon oxide. This conclusion is supported by the significant cell adhesion process observed on silicon oxide in opposition to parylene-C.

Fn is a dimeric glycoprotein, which in soluble form, is believed to have a disk-shaped globular and compact structure stabilized by intramolecular ionic interactions between domains with dimensions of 30 nm diameter and 2 nm in height [37]. Under acidic, alkaline and high ionic strength conditions, Fn has been reported to acquire a more extended form [38]. An AFM study has revealed that on hydrophilic surfaces, such as silica, most of the protein molecules have an elongated structure whereas they acquire a compact structure on hydrophobic methylated surfaces [39]. In the present study, the measured mass coverage and structural values indicate that Fn forms a compact and dense layer on parylene-C and an extended and diffuse layer on silicon oxide (Fig. 7). However, despite the structural difference in adsorbed Fn, both parylene-C and silicon oxide substrates host sub-populations of neurons and cells with fibroblast morphology, derived from the differentiation of CGR8 mES cells. The neurons extend long, branching processes that form an interconnecting network of axons and dendrites. This is healthy neuronal behavior, commonly observed on cytophilic surfaces that encourage attachment [40]. Furthermore, the formation of distinct focal adhesion complexes in fibroblast-like cells is an indicator of integrin mediated cell adhesion to the ECM [41].

### 4.2 Albumin layers

Assuming albumin dimensions in the globular state of 4 nm × 4 nm × 14 nm conferring a prolate spheroidal shape [42], the theoretical full monolayer coverage with side-on conformation corresponds to an adsorbed mass of 1.96 ng/mm^2^. The data show that saturation of the surface is reached on parylene-C but not on SiON meaning that a complete monolayer is only obtained on parylene-C. Considering the net charge of albumin of −14 at pH 7.4, electrostatic repulsion may occur between protein molecules and the negatively charged silicon oxide surface. This might explain the lower surface coverage and the easily reversible adsorption process on this substrate. The thickness value on SiON (4.3 nm) suggests that the incomplete monolayer is made of globular albumin arranged in a side-on conformation. This result is in good agreement with a previous study showing that albumin adsorbs at a mass coverage of 0.9 ng/mm^2^ on a highly hydrophilic plasma treated silica surface with a 3 nm film thickness [43]. On the other hand, the thickness observed on parylene-C, well below the dimension of the protein, indicates a strong deformation of the protein resulting from a probable unfolding of albumin on the surface, as previously suggested on a hydrophobic surface [44]. The presence of an albumin layer on parylene-C substrate prevents cell adhesion, while it allows some neuronal growth on silicon oxide. This latter observation is nonetheless significantly lower than the cell adhesion process observed in the presence of Fn and can be due to weak cell attachment.

### 4.3 Competitive adsorption of Fn and albumin

The co-adsorption of Fn and albumin at a mass ratio of 1:100 results in the formation of distinct layers depending on the substrate. Silicon oxide and parylene-C promote preferential adsorption of Fn and albumin, respectively. This conclusion is drawn from the comparison made with the layer properties of the single protein system that are similar to Fn on silicon oxide and similar to albumin on parylene-C. It appears that Fn preferentially adsorbs to silicon oxide despite the presence of a high concentration of albumin in the bulk solution. Conversely, on parylene-C, albumin outcompetes Fn.

These findings can be explained by a phenomenon referred to as the Vroman effect or competitive protein exchange that occurs when a protein mixture adsorbs to a substrate [45,46]. Low molecular weight proteins at high concentration will adsorb more readily to the substrate but will be displaced by higher molecular weight proteins [47]. Albumin adsorbed to substrates has been reported to be displaced by higher molecular weight proteins such as IgG and fibrinogen [47,48]. This protein exchange is explained by a desorption/adsorption process and the fact that higher molecular weight proteins have higher affinity for the substrate as their large and flexible structures enable more surface contacts [47,49,50].

On silicon oxide, Fn can displace albumin from the substrate as the adsorption process of albumin is reversible. In contrast, on parylene-C, albumin adsorbed first and irreversibly, prevents any exchange with Fn. In accordance with our data, a reduced ability of Fn to displace albumin was observed on a hydrophobic substrate [51].

However, preferential adsorption of Fn onto hydrophobic interfaces in presence of albumin has previously been reported by Vaidya et al. and Giamblanco et al. [52,53]. Giamblanco and Vaidya used Fn/albumin mixtures of 1:1 and 1:25, respectively (mass ratios) to study competitive protein co-adsorption on hydrophobic interfaces. The mass ratios they used are substantially different from the one we used (1:100), which is also the mass ratio found in physiological conditions (serum).

Our conclusion that, in contrast to silicon dioxide, albumin adsorbs irreversibly on parylene-C and cannot be outcompeted by Fn is also supported by the formation of distinct focal adhesion complexes on Fn/albumin treated silicon oxide but not on parylene-C. This implies that in the former case Fn is present, but in the latter it is absent. This could also explain the lack of Fn detection on polyacrilimide gel electrophoresis eluents from parylene-C performed by Delivopoulos et al. [9].

Further investigations of serum protein adsorption and mechanisms of cell adhesion on parylene-C will enhance the tissue engineering and medical applications of this promising polymer.

## 5 Conclusions

The present study describes the surface coverage and structural properties of protein layers formed upon adsorption of two serum proteins, Fn and albumin, on two substrates presenting contrasting chemistry, silicon oxide and parylene-C. Using an optical biosensing technique, DPI, we have shown that Fn and albumin have higher adsorption on parylene-C compared to silicon oxide. Moreover, the layer properties are significantly different with formation of thin and dense layers on parlene-C and thicker and more diffuse layers on silicon oxide. These results suggest that Fn presents a compact structure on parylene-C, whereas it adopts an extended structure on silicon oxide.

Nonetheless, differentiated mES cells exhibited focal adhesion complexes on both parylene-C and silicon oxide Fn coated substrates. Albumin follows the same trend with a compact and deformed structure on parylene-C and a globular structure on silicon oxide. However albumin coated substrates do not support significant cell attachment in either state. Interestingly, a binary mixture of Fn and albumin results in a preferential adsorption process, with formation of layers mainly composed of albumin on parylene-C and Fn on silicon oxide. This is further supported by the presence of focal adhesions in cells on silicon oxide, but not on the parylene-C. These results agree with our previous study on neuronal and glial cell patterning and suggest the involvement of other serum proteins in this process. The comprehensive description of serum protein adsorption and layer properties on parylene-C will elucidate the underlying material–protein–cell interactions on this substrate and enable further medical applications of this promising polymer.
