# Supplementary material for: Serum protein layers on parylene-C and silicon oxide: Effect on cell adhesion
Source: Colloids Surf B Biointerfaces. 2015 Feb 1;126:169–77. doi: 10.1016/j.colsurfb.2014.12.020 (PMC4342411; doi:10.1016/j.colsurfb.2014.12.020)

Supplementary material

Table S1. Mass coverages of albumin adsorbed on SiO_2 and SiON (concentration of 0.1 mg/ml at pH 7).

**Surface material**	**Mass (ng/mm^2)**
**SiO_2**	1.51±0.05
**SiON**<br>89% SiO_2<br>11% of silicon nitride	1.44±0.09

Table S2. Protein layers characterization on parylene-C and SiON for Fn 50 μg/mL, albumin 5000 μg/mL and a mixture of Fn 50 μg/mL and albumin 5000 μg/mL in PBS at 20°C.

**Proteins**	**Parylene-C**			**SiON**		
	**Mass (ng/mm^2)**	**Thickness (nm)**	**Density (g/mL)**	**Mass (ng/mm^2)**	**Thickness (nm)**	**Density (g/mL)**
**Maximum adsorption**						
**Fn**	2.51±0.06	3.2±0.2	0.79±0.05	2.06±0.04	8.9±1.4	0.23±0.04
**Albumin**	1.5±0.1	1.72±0.06	0.88±0.04	0.8±0.1	4.3±0.6	0.20±0.05
**Fn/Albumin**	1.70±0.07	1.9±0.1	0.91±0.08	1.9±0.1	8.2±0.3	0.24±0.02
**Post adsorption (5 min rinsing)**						
**Fn**	2.50±0.08	3.1±0.2	0.81±0.05	1.89±0.03	7.5±0.9	0.25±0.03
**Albumin**	1.5±0.1	1.80±0.06	0.85±0.06	0.66±0.05	3.2±0.6	0.21±0.04
**Fn/Albumin**	1.69±0.07	1.89±0.07	0.90±0.05	1.7±0.1	6.9±0.2	0.24±0.02

Fig. S1. AFM morphology analysis of silicon oxide surface before (A) and after (B) parylene-C coating). (B) Image of a scalpel blade incision in the deposited parylene-C layer. The silicon oxide surface underneath the parylene-C is revealed and the step between the two layers is measured at approximatively 30 nm.

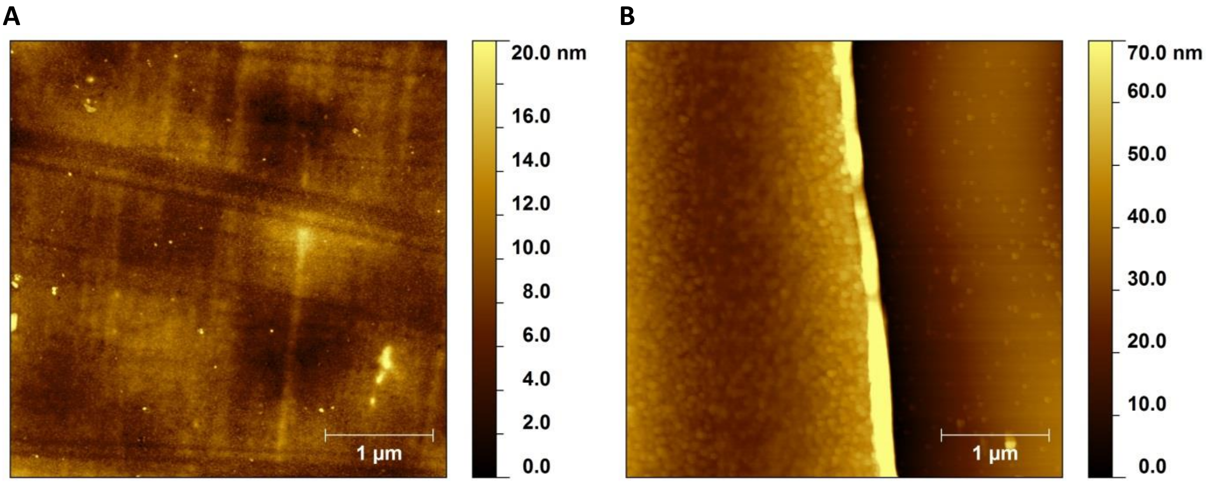

Supplement: Supplementary file 1 [file mmc1.pdf]
